# A pangenome insight into the genome divergence and flower color diversity among *Rhododendron* species

**DOI:** 10.1186/s12864-025-12461-5

**Published:** 2026-01-06

**Authors:** Hai-Yao Ma, Shuai Nie, Hai-Bo Liu, Tian-Le Shi, Shi-Wei Zhao, Zhao-Yang Chen, Yu-Tao Bao, Zhi-Chao Li, Jian-Feng Mao

**Affiliations:** 1 State Key Laboratory of Tree Genetics and Breeding, National Engineering Research Center of Tree Breeding and Ecological Restoration, National Engineering Laboratory for Tree Breeding, Key Laboratory of Genetics and Breeding in Forest Trees and Ornamental Plants, Ministry of Education, College of Biological Sciences and Technology, Beijing Forestry University, Beijing, 100083 China; 2 Rice Research Institute, Guangdong Key Laboratory of New Technology in Rice Breeding, Guangdong Rice Engineering Laboratory, Key Laboratory of Genetics and Breeding of High Quality Rice in Southern China (Co-construction by Ministry and Province), Ministry of Agriculture and Rural Affairs, Guangdong Academy of Agricultural Sciences, Guangzhou, 510640 China; 3 Jinan Academy of Landscape and Forestry Science, Jinan, 250100 China; 4 Institute of Forestry and Pomology, Beijing Academy of Agriculture and Forestry Sciences, Beijing, 100093 China; 5 Umeå Plant Science Centre, Umeå University, Umeå, SE-901 87 Sweden

**Keywords:** Rhododendron, Transposable element, Gene duplication, Gene loss, Flower color

## Abstract

**Background:**

The *Rhododendron* genus (*Rhododendron* L.), recognized as the most extensive woody plant genus in the Northern Hemisphere, captivates with its strikingly beautiful corollas and variety of flower colors. In addition, the *Rhododendron* genus exhibits a complex evolutionary history and substantial species diversification. To comprehensively understand the genomic complexity and flower color diversity within this genus, comparative genomics has emerged as a promising approach, enabling analysis at a super-species level.

**Results:**

Here, we collected whole-genome data from seven rhododendrons of different subgenera to investigate the patterns of interspecific genomic and sequence divergence, as well as evolutionary dynamics of gene family related to flower color. We discovered that approximately 50% of *Rhododendron* genomes are composed of transposable elements (TEs), with over half of them being long terminal repeat retrotransposons (LTR-RTs). TEs significantly associate with genomic differentiation and structural variances within the genus. Additionally, the duplication and loss of genes associated with flower color and their corresponding expression over time are potentially driven by TEs.

**Conclusion:**

Our comparative genomic analysis accentuates the critical role of TEs in genome divergence within the *Rhododendron* genus, highlighting their potential role as a key factor governing speciation and interspecific variability within the genus.

**Supplementary Information:**

The online version contains supplementary material available at 10.1186/s12864-025-12461-5.

## Background

*Rhododendron* L., represents one of the largest groups of woody plants globally and the largest genus of woody plants in China [1–4]. With nearly 1,000 species, *Rhododendron* is widely distributed in the Northern Hemisphere, mainly in Asia, Europe, and North America [5]. In China, it is known as the king of woody flowers due to its stunning diversity of colors.

Rhododendrons are characterized by their complex evolutionary history, widespread taxa, and abundant flower diversity resources. Due to recent rapid radiations and widespread hybridization, the genus identifies a large number of morphologically diverse species, and its taxonomy has undergone several major revisions [6, 7]. This complex evolutionary history has resulted in extensive genomic complexity in the genus, including significant variations in genome size, proportion of repetitive sequences, and gene number. However, our current understanding and comprehensive characterization of the genomic complexity among *Rhododendron* species are still limited.

*Rhododendron* species display a wide range of flower color variations, such as red, crimson, purple, yellow, and white [8]. The genomic assemblies of *Rhododendron* species with diverse flower colors in red (including pink), yellow, and white, have already been published. Flower color diversity among rhododendrons is primarily determined by flavonoid compounds, particularly anthocyanins [2, 9]. Previous studies have shown that the anthocyanin biosynthesis genes in *Rhododendron* experience drastic duplicates and losses [10, 11]. Abundant tandem duplication (TD) and proximal duplication (PD) genes and pseudogenes are found within the anthocyanin biosynthesis gene families, which may explain the absence of pelargonidin biosynthesis in *R. molle* flowers [10]. However, further studies with larger sample sizes are needed, as existing studies have only been conducted on a small number of *Rhododendron* species with certain flower colors. The complex flower color variation in *Rhododendron* provides great potential for exploring flower color evolution.

The advancements in genomics, represented by comparative genomics, have enabled researchers to simultaneously study the sequence characteristics of species at both the whole-genome and gene levels [12]. In the *Rhododendron* genus, multiple high-quality genome assembly projects have been reported, providing opportunities to investigate the connection between genomic complexity and flower color diversity among species. Previous studies on individual genomes have found that transposable elements (TEs), particularly long terminal repeat retrotransposons (LTR-RTs), play a significant role in driving genome and gene variation, thereby potentially trigger phenotypic variation. The maize color variety is a well-known example of how TE insertion can affect individual attributes [13]. Sunflowers also exhibit substantial genetic variation due to TEs, particularly LTR-RTs [14]. Numerous studies have also revealed a strong association between TEs and pseudogenization, the loss of functional gene. In Triticeae species, TE insertion and TE-driven gene mobility have generated thousands of pseudogenes, facilitating rapid differentiation of the Triticeae genomes [15]. In *R. molle*, the insertion of LTR-RTs has led to the expansion and contraction of the CHS gene family, which is closely related to flower color variation [10]. However, there is currently a lack of interspecific comparisons of more genomes to further investigate this phenomenon. Currently, our knowledge of TE-related phenotypic variation among species within the same genus remains limited, with most research focused on large-scale genomic projects and model species [16].

This study aims to investigate the genomic divergence among *Rhododendron* species, and further to discover the potential impact of TEs on genome variation and phenotypic. Additionally, given that flower color holds significant value as an ornamental trait within the *Rhododendron* genus, this study annotated genes associated with the biosynthesis of major pigments such as anthocyanins, chlorophyll, and carotenoids. Gene gain and loss dynamics were also revealed, shedding light on the functional significance of TEs in shaping genomic variation among *Rhododendron* species. This research enhances our understanding of the functional role of TEs in genome divergence within the *Rhododendron* genus, and lays the foundation for further exploring the driving force of TEs in phenotypic diversity.

## Materials and methods

### Data collection

We collected publicly released whole genome data of nine *Rhododendron* species, including *R. molle* (yellow flowers) [10], *R. simsii* (red flowers) [11], *R. griersonianum* (red flowers) [17], *R. ovatum* (white to pink flowers) [18], *R. ripense* (purple flowers) [19], *R. henanense* (white flowers) [20], *R. prattii* (white flowers) [21], *R. williamsianum* (pink flowers) [22] and *R. delavayi* (red flowers) [23]. The genome sequences, protein sequences, annotation files, and CDS sequences were obtained from databases such as NCBI, NGDC, GigaDB, and others (see Supplemental Table 1 for details on data origin). We specifically selected seven *Rhododendron* species with high-quality genome and chromosome-level assemblies for subsequent in-depth analyses.

### Phylogenomic analyses for Ericales

A total of 17 species were selected to reconstruct the evolutionary history of the Ericales clade, with *Nyssa sinensis* [24] as an outgroup. Orthogroups were constructed by selecting the longest transcript for each coding gene using OrthoFinder v2.3.1 [25]. A total of 243 single-copy and 1,513 low-copy orthogroups (with at least 88.2% of species having single-copy genes in any orthogroup) were extracted. Amino acid sequences from each orthogroup were aligned using MAFFT v7.471 [26], and then PAL2NAL v.14 [27] was used to convert the protein alignments into codon-preserving alignments.

Gene trees for individual low-copy orthogroups were constructed using 1,000 bootstrapping replicates, and each node of the gene trees was checked with the bootstrap support (BS) cutoff value of 50%. Phylogenetic inference was performed using ASTRAL-Pro v1.1.2 [28] based on the gene trees. We also used 989 low-copy orthogroups (with at least 90% of the species having single-copy genes in any orthogroup) to construct a species tree using the above method in order to obtain a more accurate and high-supporting species relationship. Finally, a consistent topology was inferred with high BS values and LPP (local posterior probability) values.

The divergence times were estimated with the MCMCTree module in the PAML package [29]. The age of the phylogenic tree was calibrated using fossils of crown nodes of Ericales (89 Mya) and *Rhododendron* (56 Mya), and the timing of the tree root (105 Ma) was calibrated using the TimeTree online database (http://www.timetree.org/).

### Genomic repeats annotation

The PanEDTA pipeline [30] was used to annotate and classify transposable elements for seven *Rhododendron* species with chromosome-scale genome assembly. BEDTools v2.30.0 [31] was used to calculate the proportion of transposable elements (TEs) in the genome.

To more accurately predict long terminal repeat retrotransposons (LTR-RTs), LTRharvest [32] and LTRdigest [33] within the genometools v1.5.10 software [34] were used for *de novo* prediction of LTR-RTs. LAST v983 (http://last.cbrc.jp) was employed to align RT sequences of LTR-RTs to the REXdb v3.026 [35] database. The resulting alignments were then used to classify identified LTR-RTs based on the completeness of their internal structure. LTR-RTs that contained a complete Gag-Pol protein sequence were defined as intact LTR-RTs (*I*). Using these intact LTR-RTs as references, blastn was used to identify homologous sequences from the genome sequence. LTR-RTs sequences with no homologous hits in both upstream and downstream regions were defined as solo LTR-RTs (*S*), while those with homologous hits on one side but not the other were defined as truncated LTR-RTs (*T*). The identified intact LTR-RTs were mainly classified into the *Copia* and *Gypsy* superfamilies based on the order and similarity of their Pol protein domains.

After counting the numbers of intact, truncated, and solo LTR-RTs, the values of S: I, T: I, (S + T): I, and S + T + I were calculated based on these statistics to roughly measure the birth and elimination rates of LTR-RTs. To eliminate the effect of assembly sequence length on the calculation of these ratios, different assembly sequence length filtering thresholds were used until the S: I ratio reached stability. In addition, multiple ratios of S, T, and I at the level of LTR-RTs families were also calculated, and the proportion of families with an S: I ratio greater than 3 was used to evaluate the elimination ability of LTR-RTs.

Finally, we utilized SubPhaser [36] to identify species-specific k-mers and thus obtain those high-copy number (copy number > = 200) and species-specific LTR-RTs from the seven *Rhododendron* species.

### Genome comparison

We performed the pairwise alignment among seven *Rhododendron* species with chromosome-scale genome assembly available using minimap2 v2.17 (-ax asm5 -k 17) [37], and then observed the synteny blocks revealed in the dotplots to determine the pairwise homology of chromosomes. As it has the highest quality in genome assembly, so *R. molle* genome was used as the reference in this study, and others’ genomes were used as queries for whole genome alignment. Then, SYRI v1.3 [38] was used to perform comparison between the reference and queries. This enabled us to identify both local (SNPs and short indels) and structural variations (inversions, translocations, and duplicates, etc.) among genomes.

The number of overlaps between LTRs and inversion, collinear, and specific regions in the genome was calculated using BEDtools v2.30.0 [31]. Here, LTRs include both randomly generated and observed LTRs. The Random function in BEDtools v2.30.0 [31] was used to generate random data. To investigate the impact of LTRs on interspecies structural variation, we compared the number of overlaps between LTRs and the inversion regions (INV), species-specific regions (SPE), and species-syntenic regions (SYN) in seven *Rhododendron* species.

### Species-specific LTR identification

For the identification, classification, and insertion age estimation of species-specific LTR-RTs, we used SubPhaser [36]. Initially, k-mers that differentiate these genomes were identified. LTR-TRs were then recognized using LTRharvest v.1.6.1 [32] and LTRfinder v.1.07, and subsequently classified with TEsorter. The insertion time (T) was calculated using the formula T = K/2r, where *r* = 1.3 × 10^–8^ substitutions yr–1 (default) and K represents the sequence divergence estimated between two LTRs from an intact LTR-RT [39].

### Gene expression

We explored the associations between interspecific gene expression differences and genomic divergence. Transcriptome data from five developmental stages of *R. mole* and *R. simsii* were collected to study interspecific differences in gene expression (Supplementary Fig. 6). Based on the comparative genomic results, genes overlapping with either specific or syntenic regions were counted. Genes overlapping with both regions were manually identified using IGV v2.16.0 [40]. And the genes that were not assigned to either cluster were not discussed in this study due to their relatively small quantity. The gene expression levels between specific regions and syntenic regions were also compared.

### Flower color genes

Enzymatic genes from the biosynthetic pathways of major pigments (here, anthocyanins, carotenoids, and chlorophylls) determining flower color were annotated with the Ensemble Enzyme Prediction Pipeline (E2P2) package v3.1 (https://gitlab.com/rhee-lab/E2P2) [41], for each whole genome seven *Rhododendron* species. Metabolic pathways were followingly constructed with reference to PlantCyc (https://plantcyc.org/) based on the annotated protein sequences.

In this study, two types of gene duplicates were defined based on the following rules: genes physically located adjacent to each other on the same chromosome were defined as tandem duplication (TD) genes, while a pair of homologous genes located on the same chromosome and separated by 1 to 10 genes were defined as proximal duplication (PD) genes. Then the TD genes and PD duplication could be identified in the enzymatic genes were manually identified and counted.

### Identification of pseudogenes

In this study, pseudogene sequences were identified and annotated using the default settings of the PseudoPipe pipeline [42], providing information on their chromosomal positions, sequences, and parent genes (i.e., homologous coding genes). To obtain high-quality pseudogene identification, the following filtering criteria were employed: (1) The sequence similarity in amino acid sequences between the pseudogene and parent gene should be greater than 30%; (2) The parent gene should cover more than 50% of the pseudogene.

### Micro-collinearity in the flower color gene family

First of all, the gene counts and the proportion of TD and PD genes within gene families related to the biosynthesis of anthocyanins, chlorophylls, and carotenoids were counted. Among these gene families, the CHS gene family exhibited a higher gene count, with a substantial presence of TD and PD genes. We further explored the CHS gene family across different species of rhododendrons. Subsequently, these TD and PD genes were positioned on chromosomes. And the homologous chromosomal segments from the seven *Rhododendron* species with the highest gene counts were selected for visualization. BEDTools was employed to extract the seven homologous LTR-RTs surrounding these CHS genes. Finally, Muscle [43] was utilized to construct a phylogenetic tree for these TD and PD genes. Based on the subfamilies of these LTR-RTs and the phylogenetic tree of the CHS enzyme genes, collinearity relationships of these genes were inferred and visualized.

## Results

### TEs contribute to genome size variation

**Fig. 1:**
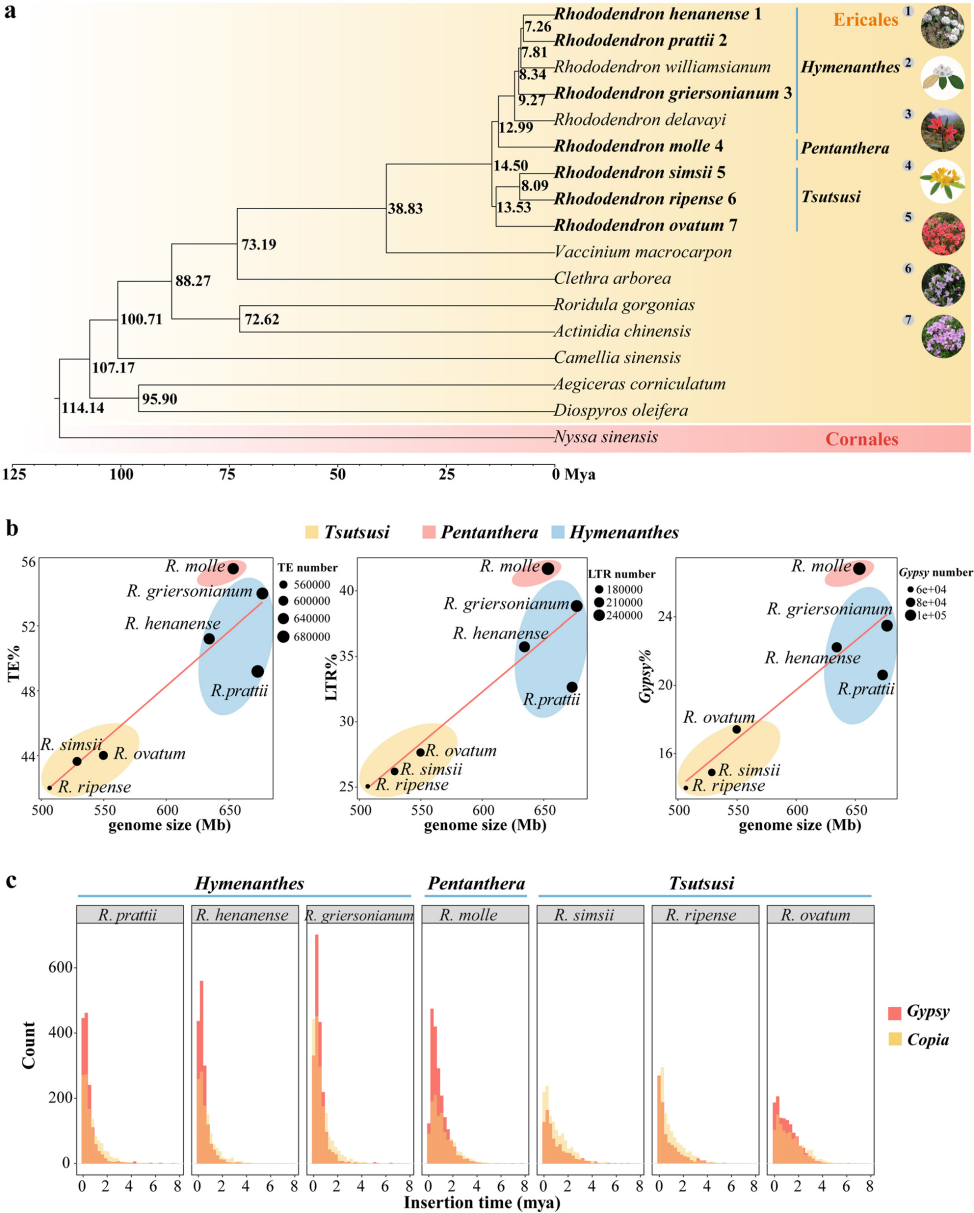
Transposable elements (TEs) and genomes size differentiation among seven *Rhododendron* species. **a** Phylogenetic tree showing divergence times in Ericales and Cornales. The numbers represent divergence time of each node (Mya, million years ago), and the species names in bold represent the focal *Rhododendron* species studied in this article, as illustrated on the right side of the figure (*R. henanense* [20], *R prattii* [21], *R. griersonianum* [17], *R. molle* [21], *R. simsii* [18], *R. ripense* [44], *R. ovatum* [18]). **b** Correlation between genome sizes and the proportion of TEs, LTR-RTs and *Gypsy* elements in the genomes. And the size of dot represents the number of TE, LTR-RT and *Gypsy*. **c** The insertion time of intact *Copia* and *Gypsy* in genomes of *R prattii*,* R. henanense*, *R. griersonianum*, *R. molle*, *R. simsii*, *R. ripense*, *R. ovatum*, respectively

The constructed phylogenetic tree divides the *Rhododendron* species studied into three distinct groups consistent with the treatment of subgenera: the *Hymenanthes* (*R. henanense*, *R. prattii* and *R. griersonianum*), the *Pentanthera* (*R. Molle*) and the *Tsutsusi* subgenus (*R. ripense*, *R. ovatum*, and *R. simsii*) (Fig. 1a). Among these seven *Rhododendron* species, genome size is varied, ranging from 506.73 Mb (*R. ripense*) to 676.81 Mb (*R. griersonianum*) (Supplementary Table 1). Species with closely related phylogenetic relationships display minimal variation in genome sizes (Fig. 1a and Supplementary Table 1). For example, the *Tsutsusi* subgenus species (*R. ripense*, *R. ovatum*, and *R. simsii*) have relatively small genome sizes. A significant amount of TEs was found in *Rhododendron* genomes, with approximately half of the genomes composed of TEs across all species (42.00% (212.82 Mb)-55.51% (362.74 Mb)) (Table 1). We found a positive correlation between genome size and TEs content (Fig. 1b). *Rhododendron* species with larger genomes had higher proportions and quantities of TEs, while the *Tsutsusi* subgenus, characterized by smaller genomes, had a lower TE content (Fig. 1a and b). In all *Rhododendron* species, the most abundant classified TE was always LTR-RT, accounting for more than half of the total annotated TEs (59.66%-75.12%) (Table 1). We observed a low proportion of novel LTR-RTs (specific to one species) in seven *Rhododendron* species, indicating a high degree of shared evolutionary history among them (Table 1). At the superfamily level, the contribution of the *Copia* elements was similar across the seven genomes (3.60%-4.54%), while *Gypsy* elements exhibited significant interspecific variation and were much more prevalent than *Copia* (13.99%-26.82%) (Table 1). LTR-RTs and *Gypsy* elements show a positive correlation with genome size and were substantially more prevalent in the *Hymenanthes* and *Pentanthera* subgenera compared to the *Tsutsusi* subgenus (Fig. 1b). Therefore, both the overall TE abundance and LTR-RTs content exhibit distinct phylogenetic signals.

**Table 1:** The proportion of various transposable elements (TEs) in the genomes of seven *Rhododendron* species

Class	*R*. *prattii*	*R*. *henanense*	*R*. *griersonianum*	*R*. *molle*	*R*. *simsii*	*R*. *ripense*	*R*. *ovatum*
LTR (%)	32.65	35.74	38.84	41.70	26.20	25.06	27.65
novel LTR (%)	0.02	0.03	0.00	0.04	0.03	0.01	0.01
LTR *Copia* (%)	3.66	3.60	4.51	3.76	4.54	4.28	3.67
novel LTR *Copia* (%)	0.01	0.00	0.00	0.02	0.00	0.00	0.00
LTR *Gypsy* (%)	20.61	22.23	23.49	26.82	14.90	13.99	17.42
novel LTR *Gypsy* (%)	0.01	0.01	0.00	0.00	0.01	0.00	0.01
LTR unknown (%)	9.37	11.03	12.17	12.91	7.53	7.55	7.43
novel LTR unknown (%)	0.01	0.02	0.00	0.01	0.01	0.01	0.00
MITE (%)	5.33	5.16	4.62	4.38	6.09	5.96	5.75
novel MITE (%)	0.04	0.01	0.01	0.01	0.01	0.03	0.01
DNA (%)	12.39	11.50	11.63	10.44	12.75	12.28	11.90
novel DNA (%)	0.76	0.66	0.52	0.35	0.52	0.41	0.50
Helitron (%)	0.49	0.49	0.45	0.42	0.47	0.46	0.47
novel Helitron (%)	0.29	0.22	0.11	0.06	0.16	0.19	0.19
total TE (%)	49.19	51.20	53.98	55.51	43.64	42.00	44.01
novel total TE (%)	0.82	0.70	0.53	0.39	0.56	0.45	0.51
genome size (Mb)	673.07	634.29	676.81	653.46	528.64	506.73	549.71

**“**novel**”** means high copy number and specific-specific TEs identified with the SubPhaser pipeline [36] in any one of the seven species

### *Gypsy* dominated evolution of TEs and genome size differentiation

The differential proportion of TEs among these seven species may be attributed to the variations in LTR-RTs overall generation capacity. LTR-RTs can be categorized into Intact LTR-RT (*I*), Solo-LTR (*S*), and Truncated LTR (*T*), and the birth and elimination rates can be assessed and ranked based on their abundance. Species in *Rhododendron* have markedly varied capacities for LTR-RTs generation and elimination, possibly related to differences in genome size. For instance, *R. griersonianum* has the highest generation rate, but its elimination rate is lower than *R. molle* and *R. prattii*, resulting in the largest genome size (Table 2). To investigate the source of interspecific variation in genome size caused by LTR-RTs, further analysis was conducted on their superfamilies. At the individual LTR-RT superfamily level, distinct abundances are observed among species (Table 3). Notably, among all the LTR *Gypsy* and *Copia* superfamilies in the *Hymenanthes* and *Pentanthera* subgenera, Retand has the highest representation in the genome (Table 3 and Supplementary Fig. 1). In the *Tsutsusi* subgenus, although Retand does not have the highest proportion, its content significantly differs from other subgenera of *Rhododendron* (Table 3).

**Table 2:** The birth and death of long terminal repeat retrotransposons (LTR-RTs) in seven *Rhododendron* species

Species	Intact LTR-RT (I)	Solo-LTR (S)	Truncated LTR (T)	S + T	S + T + I	SUM of All Modules	Filtered scaffold length (bp)	Filtered I	Filtered S	Filtered T	Filtered S/I	Filtered T/I	Filtered (S + T)/I	S/I > 3ModuleSUM	S/I > 3ModuleSUM/AllModuleSUM
*R. prattii*	2,656	21,281	2,523	23,804	26,460	401	805,000	2,474	19,238	2,308	7.78	0.93	8.71	202	50.37%
*R. henanense*	2,770	15,452	1,490	16,942	19,712	407	1,995,000	2,750	15,337	1,480	5.58	0.54	6.12	198	48.64%
*R. griersonianum*	3,775	28,673	3,487	32,160	35,935	454	1,455,000	3,714	27,950	3,403	7.53	0.92	8.44	216	47.57%
*R. molle*	3,239	25,954	3,751	29,705	32,944	561	335,000	3,230	25,930	3,748	8.03	1.16	9.19	267	47.59%
*R. simsii*	2,096	10,360	1,878	12,238	14,334	389	900,000	1,880	9,067	1,593	4.82	0.85	5.67	172	44.21%
*R. ovatum*	2,394	8,806	1,565	10,371	12,765	474	0	2,394	8,806	1,565	3.68	0.65	4.33	232	48.94%
*R. ripense*	2,208	8,236	1,438	9,674	11,882	378	2,245,000	2,071	7,815	1,354	3.77	0.65	4.43	149	39.41%

“*S*/*I* > 3ModuleSUM” represents the module that maintains high removing rate when the ratio of solo LTR-RTs to intact LTR-RTs is more than 3. The ratio “*S*/*I* > 3 ModuleSUM/AllModuleSUM” represents the proportion of modules with high removing rate in the total number of modules

**Table 3:** The proportion of long terminal repeat-retrotransposons (LTR-RTs) superfamilies in the genomes of seven *Rhododendron* species

	*Hymenanthes*			*Pentanthera*	*Tsutsusi*		
	*R*. *prattii*	*R*. *henanense*	*R*. *griersonianum*	*R*.* molle*	*R*. *simsii*	*R*. *ripense*	*R*. *ovatum*
*Copia*	21.28%	20.80%	24.14%	18.30%	37.83%	38.19%	26.09%
*Copia Ale*	8.15%	7.71%	7.97%	7.45%	12.93%	14.75%	9.05%
*Copia Alesia*	0.00%	0.01%	0.04%	0.05%	0.02%	0.00%	0.00%
*Copia Angela*	2.12%	2.21%	2.87%	1.40%	5.38%	5.75%	1.84%
*Copia Bianca*	0.22%	0.21%	0.52%	0.46%	0.20%	0.26%	0.66%
*Copia Ikeros*	0.47%	0.49%	0.65%	1.43%	2.55%	1.85%	1.97%
*Copia Ivana*	1.29%	0.81%	0.69%	1.24%	1.90%	2.23%	1.57%
*Copia SIRE*	1.48%	1.27%	1.44%	1.39%	1.77%	2.20%	1.98%
*Copia TAR*	2.43%	2.71%	3.53%	2.18%	5.55%	5.00%	3.89%
*Copia Tork*	4.26%	4.51%	5.33%	2.05%	6.15%	4.98%	4.14%
*Gypsy*	47.85%	51.20%	45.96%	52.08%	40.12%	43.38%	53.24%
*Gypsy Athila*	11.36%	12.04%	10.95%	12.51%	12.61%	12.22%	15.13%
*Gypsy CRM*	1.17%	1.53%	2.39%	1.78%	3.12%	2.50%	2.30%
*Gypsy Galadriel*	0.03%	0.05%	0.10%	0.17%	0.10%	0.06%	0.20%
*Gypsy Ogre*	8.90%	8.15%	8.34%	8.94%	8.17%	8.90%	10.55%
*Gypsy Reina*	0.17%	0.13%	0.06%	0.12%	0.32%	0.37%	0.33%
*Gypsy Retand*	20.06%	21.68%	15.52%	19.07%	5.76%	6.15%	10.03%
*Gypsy TatIII*	0.00%	0.01%	0.00%	0.00%	0.00%	0.00%	0.00%
*Gypsy Tekay*	5.51%	7.02%	7.39%	8.45%	9.13%	12.26%	13.15%
Unclassified	1.51%	1.48%	2.33%	1.69%	2.29%	2.11%	2.53%
genome size (Mb)	673.07	634.29	676.81	653.46	528.64	506.73	549.71

All seven species of *Rhododendron* exhibit a single ancient peak of LTR-RT accumulation within 2 million years ago (Mya), and the size of this peak aligns with the phylogenetic relationships within the *Rhododendron* genus (Fig. 1c). Recent outbreaks of *Gypsy* and *Copia* elements have been observed, but the peak sizes of these elements vary among different subgenera, with the *Gypsy* peaks in the *Hymenanthes* and *Pentanthera* subgenera being substantially taller than the *Copia* peaks. Additionally, in comparison to members of the *Tsutsusi* subgenus, significant expansions of the *Gypsy* element have occurred within the genomes of the *Hymenanthes* and *Pentanthera* subgenera over the past 2 Mya. It is apparent that the regional differentiation of genomes has been greatly aided by the abundance of LTR-RTs, particularly the *Gypsy* superfamily. Further analysis indicates that the dominant activity in the genomes of *Hymenanthes* and *Pentanthera* subgenus is likely mainly due to the significant activity of Retand, a *Gypsy* superfamily member (Supplementary Fig. 2 and Supplementary Fig. 3).

### LTR-RTs associated with structural variations

**Fig. 2:**
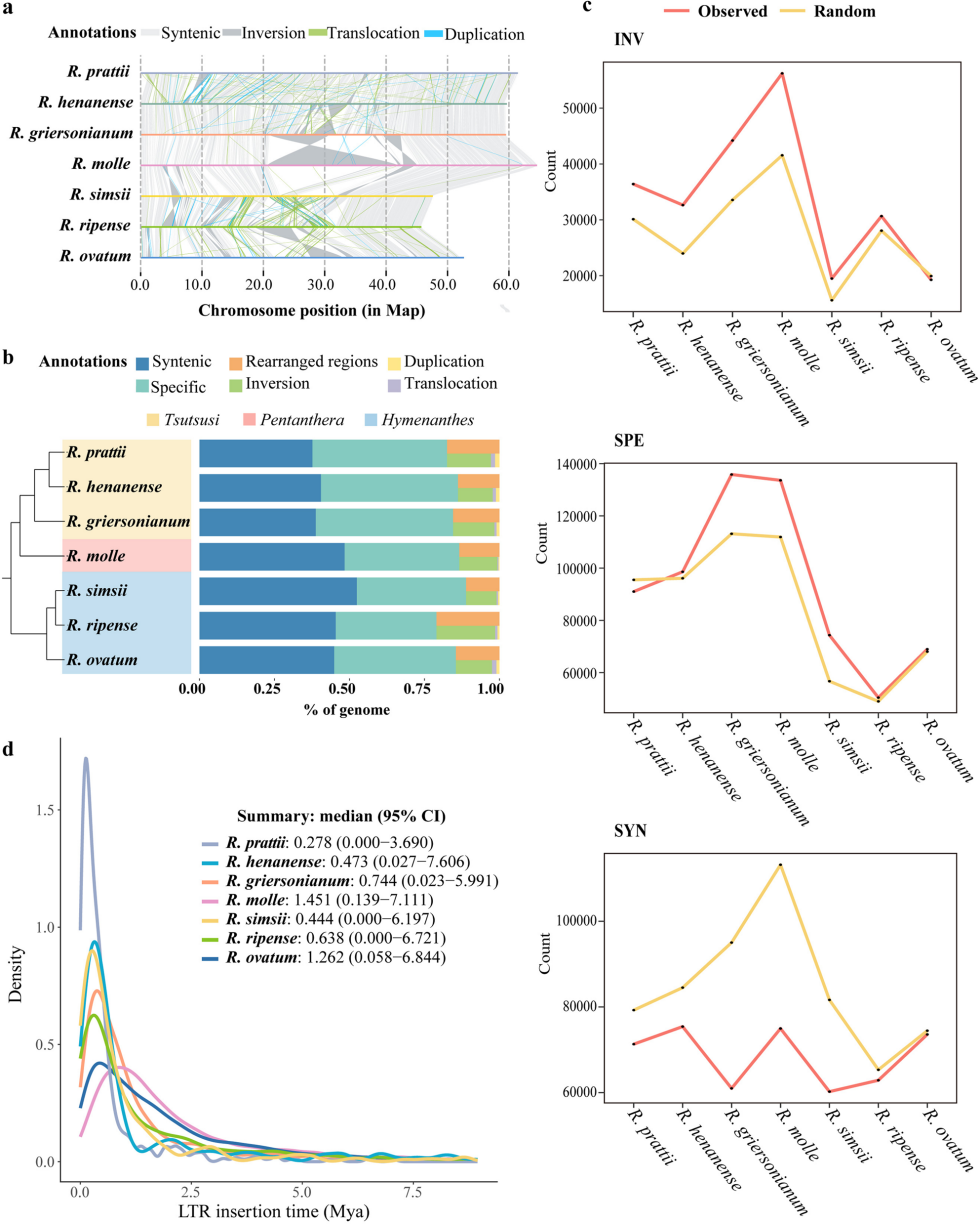
Transposable elements (TEs) and structural variation among *Rhododendron* species. **a** Structural variations detected with whole-genome alignments among the seven *Rhododendron* species (*R*. *prattii*, *R. henanense*, *R. griersonianum*, *R. molle*, *R. simsii*, *R. ripense*, *R. ovatum*). **b** Summary of structural variations. **c** The number of LTRs overlapping with structural variations in the seven *Rhododendron* species. **d** Insertion times in the seven *Rhododendron* species of LTR-RTs.

Comparative genomic analyses revealed considerable structural variation among seven *Rhododendron* species, including species-specific regions (SPE, 31.16%-45.38% of the genome), and notable inversion regions (INV, 10.15%-18.17%) (Fig. 2a and b and Supplementary Table 2). And large species-syntenic regions (SYN, 37.36%-48.38%) were maintained in the *Rhododendron* genus. The *Tsutsusi* subgenus, characterized by the lowest content of TEs, had the largest amount of SYN, whereas the other two *Rhododendron* subgenera showed a higher proportion of SPE (Fig. 2b; Table 1).

As the most abundant genomic fraction of TEs, LTR-RTs were found to be generally associated with genomic structural variations. The actual overlap of *Rhododendron* LTR-RTs with INV and SPE was significantly greater than the random distribution, while the actual overlap with SYN was significantly smaller (Fig. 2c). Furthermore, we found a substantial bias in the species-specific LTR-RTs content in 7 *Rhododendron* species, with INV (0.344%-11.361%) and SPE (0.408%-8.653%) showing significantly higher levels, whereas SYN had much lower content (0.111%-3.786%) (Table 4). In the *Rhododendron* species, the proportion of species-specific LTR-RTs in INV was found to be highest within the *Hymenanthes* and *Pentanthera* subgenera, surpassing those in SPE (Table 4). The majority of these species-specific LTR-RTs inserted in the INV and SPE of the seven *Rhododendron* species were *Gypsy*, albeit with differences in their subfamilies (Table 4). In the subgenus *Hymenanthes*, the primary subfamily was *Ogre*, in subgenus *Pentanthera*, it was *Retand*, and in subgenus *Tsutsusi*, there were significant insertions of *Ogre*, *Retand*, and *Tekay* (Table 4). Furthermore, the insertion times of these species-specific LTR-RTs varied among the seven *Rhododendron* species, with their insertion orders largely consistent with the *Rhododendron* inter-species differentiation sequence (Figs. 1a and 2d). The majority of LTR-RTs were recent insertions and occurred after the divergence of these *Rhododendron* species (~ 7 Mya) (Figs. 1a and 2d).

**Fig. 3:**
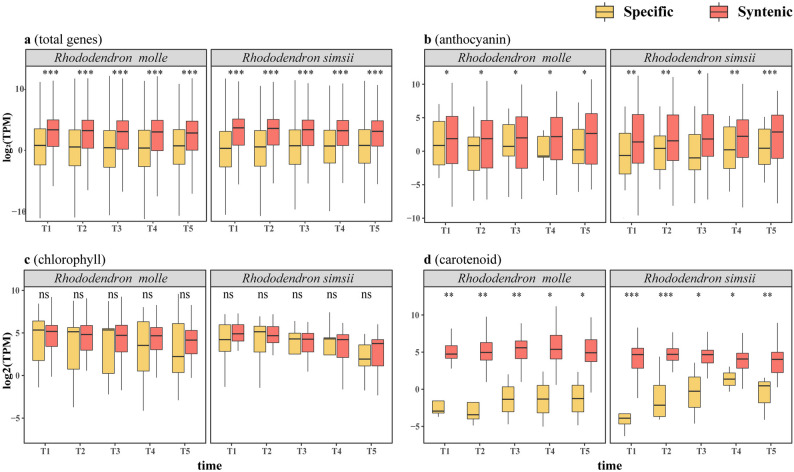
Differential expression between pigment biosynthetic genes from specific and syntenic regions. **a** Differential expression of all pigment (anthocyanin, chlorophyll, carotenoid) biosynthetic genes from specific and syntenic regions identified between R. molle and R. simsii genomes. Significance of difference is tested with Student's t test. *** p < 0.001, ** p < 0.01, * p < 0.05, ns p > 0.05. Differential expression of genes from anthocyanin (**b)**, chlorophyll **(c)**, carotenoid **(d)** biosynthesis.

**Table 4:** The proportion of species-specific LTR-RTs in the inversion, specific and syntenic regions

	*R*. *prattii*			*R*. *henanense*			*R*. *griersonianum*			*R*. *molle*			*R*. *ripense*			*R*. *simsii*			*R*. *ovatum*		
Class	INV	SPE	SYN	INV	SPE	SYN	INV	SPE	SYN	INV	SPE	SYN	INV	SPE	SYN	INV	SPE	SYN	INV	SPE	SYN
*Copia*	0.000	0.005	0.002	0.000	0.006	0.000	0.162	0.243	0.076	0.280	0.442	0.255	0.113	0.047	0.055	0.033	0.015	0.000	0.106	0.219	0.198
Copia_Ale	0.000	0.001	0.002	0.000	0.000	0.000	0.011	0.048	0.018	0.150	0.138	0.100	0.044	0.007	0.034	0.000	0.005	0.000	0.017	0.050	0.044
Copia_Tork	0.000	0.002	0.000	0.000	0.000	0.000	0.120	0.134	0.048	0.018	0.076	0.019	0.000	0.000	0.003	0.000	0.000	0.000	0.022	0.031	0.029
*Gypsy*	1.696	1.219	0.221	0.531	0.403	0.081	4.187	2.979	1.280	11.070	8.203	3.331	0.833	0.657	0.647	0.311	0.454	0.198	4.457	4.069	3.588
Gypsy_Athila	0.000	0.003	0.000	0.000	0.000	0.000	0.271	0.273	0.246	0.483	0.977	0.695	0.017	0.038	0.016	0.186	0.236	0.097	0.393	0.350	0.299
Gypsy_Ogre	1.664	1.207	0.221	0.531	0.380	0.077	2.216	1.277	0.207	3.964	2.525	0.663	0.387	0.243	0.293	0.020	0.121	0.056	0.641	0.447	0.439
Gypsy_Retand	0.029	0.009	0.000	0.000	0.015	0.005	1.126	0.737	0.383	4.392	3.407	1.441	0.282	0.276	0.262	0.000	0.013	0.000	2.685	2.425	2.126
Gypsy_Tekay	0.000	0.000	0.000	0.000	0.007	0.000	0.561	0.662	0.438	2.081	1.211	0.470	0.117	0.100	0.076	0.105	0.073	0.045	0.702	0.816	0.661
Total LTR	1.696	1.227	0.222	0.531	0.408	0.081	4.350	3.227	1.363	11.361	8.653	3.588	0.946	0.704	0.701	0.344	0.469	0.198	4.563	4.288	3.786

“INV”, “SPE”, and “SYN” designate the chromosomal inversions, species specific regions, and designates syntenic blocks, respectively

### TE involved in Spatiotemporal coordination of gene expression

In this study, we observed significant differences in gene expression between interspecific syntenic regions and specific regions during five flower development stages of *R. molle* and *R. simsi* (Fig. 3a and Supplementary Fig. 6). We hypothesize that TEs in proximity to genes may influence gene expression, resulting in decreased expression levels. Interestingly, the interspecific specific regions were enriched with more TEs, particularly members of the *Gypsy* elements, while TE insertions are less frequent in syntenic regions. This provides strong evidence that TEs can indeed influence the transcriptional expression levels of genes.

**Fig. 4:**
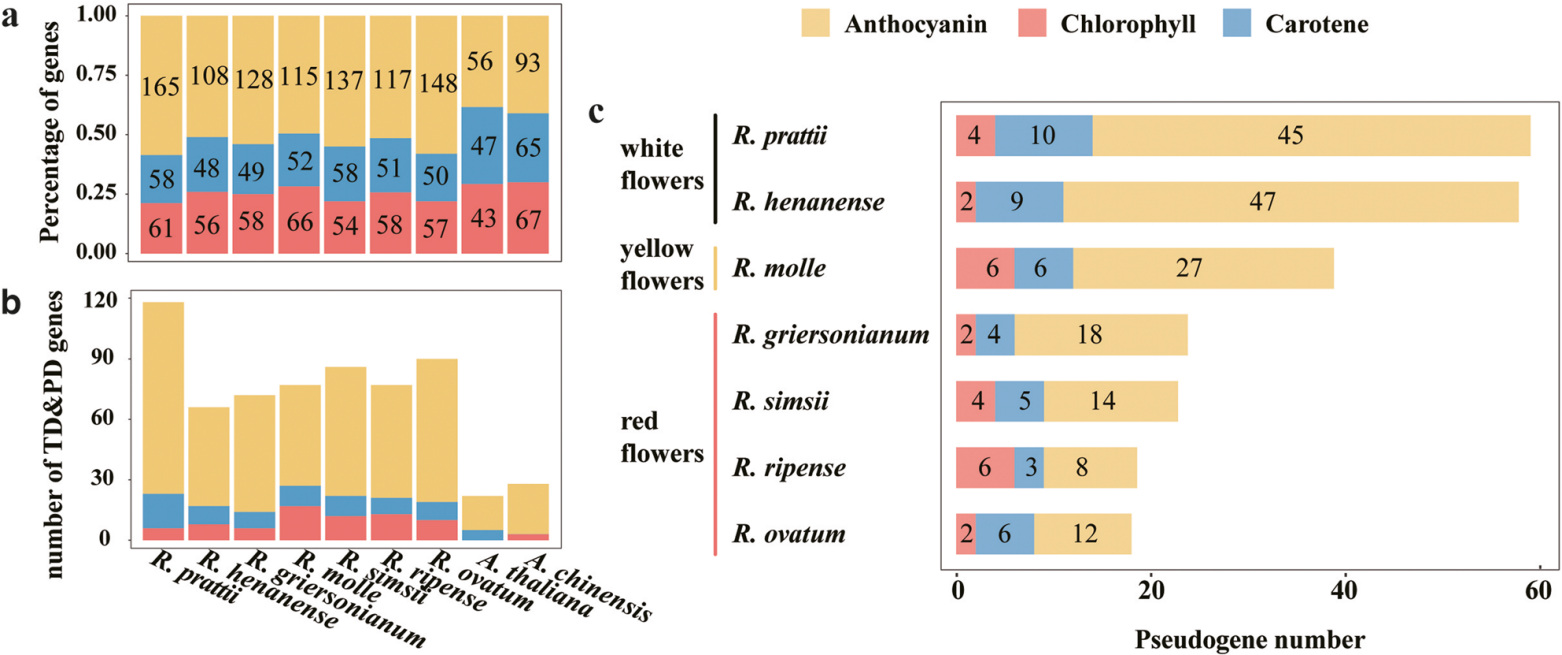
Gene duplication and loss of pigment (Anthocyanin, Chlorophyll, Carotene) biosynthesis enzymatic genes. **a** Barplot shows the counts of enzymatic gene families from different biosynthesis pathways of anthocyanin, carotenoid and chlorophyll. **b** The number of tandem duplication (TD) and proximal duplication (PD) genes from different biosynthesis pathways. **c** The pseudogene numbers from different biosynthesis pathways.

We also compared the expression levels of enzymatic genes from the biosynthesis pathways of major pigment such as carotenoid, chlorophyll, and anthocyanin between *R. molle* and *R. simsi*. In addition to chlorophyll-related genes, the pigment-related genes involved in floral coloration (anthocyanins and carotenoids) demonstrate a remarkable difference in gene expression within interspecific syntenic regions as compared to specific regions (Fig. 3b-d). These genes may play a key role in determining the color of the flowers in the five different stages of flower development and the variations in their transcriptional expression levels could potentially be influenced by TEs.

**Fig. 5:**
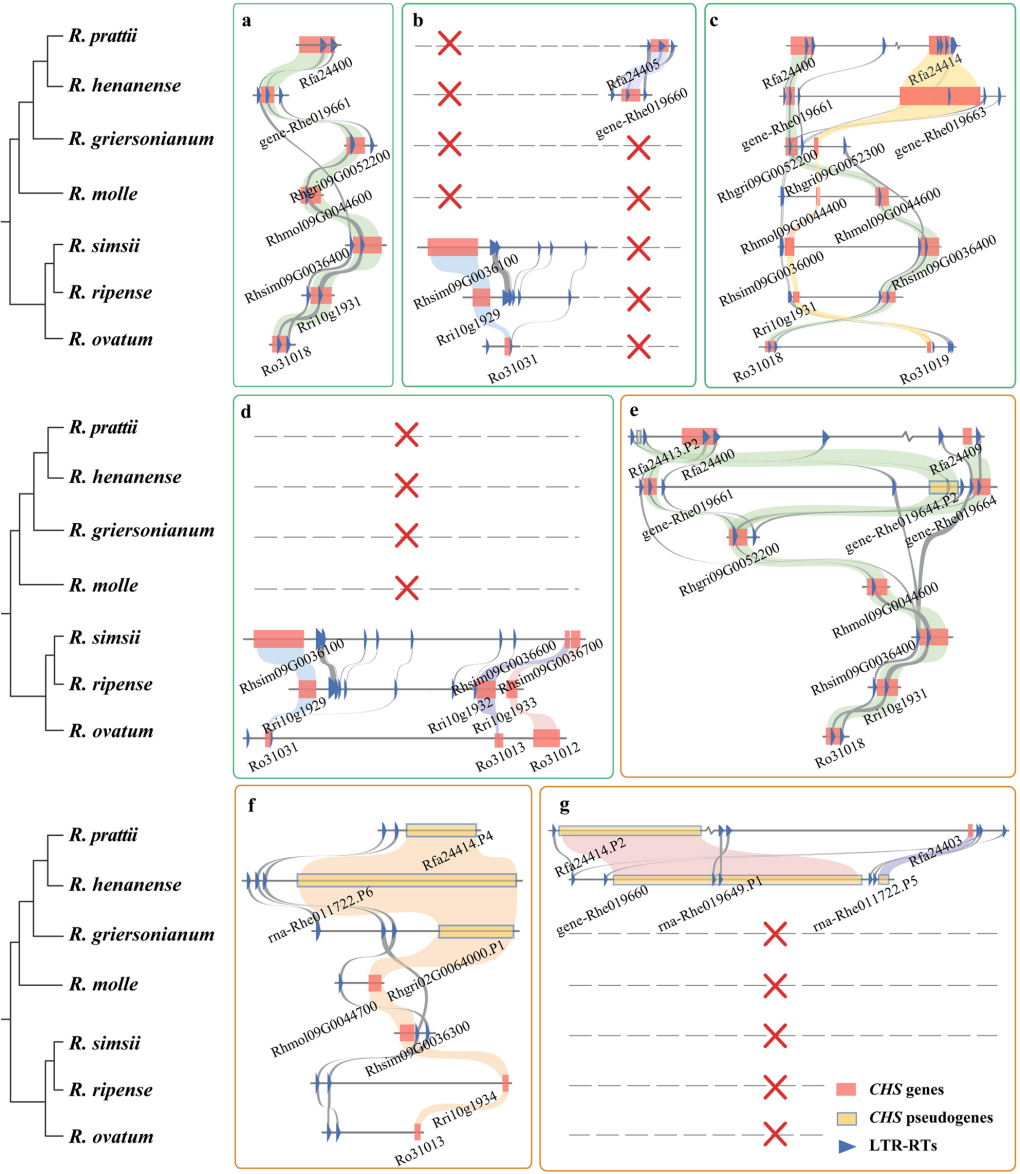
Micro-synteny of CHS genes in seven *Rhododendron* species and the resolved gene duplication and loss events. Here blue triangles represent LTR, red squares represent TD and PD genes, and yellow squares represent pseudogenes. And the grey dashed line represents the absence of syntenic genes in the corresponding species. **a** A gene is present containing a conserved LTR-RTs element (00000757 LTR unknown) in all seven species. **b **a conserved gene was found in *R. prattii* and *R. henanense*, and contained a conserved LTR-RTs element (TE_00010703 LTR/Copia) (purple). And three species from the *Tsutsusi *subgenus have a specific synteny gene (blue). **c** two CHS TD/PD genes show conservation in all seven *Rhododendron* species. **d** Three CHS genes show specific synteny in the Tsutsusi subgenus. **e** A CHS gene exhibiting collinearity across all *Rhododendron* species undergoes specific amplification in *R. prattii* and *R. henanense*, followed by pseudogenization. **f** A CHS gene showing synteny in the *Tsutsusi* and *Pentanthera* subgenera becomes a pseudogene in the *Hymenanthes* subgenus. **g** Two specific CHS gene in *R. prattii* and *R. henanense* become pseudogenes.

### TEs’ potential role in gene duplication and pseudogenization

The number of enzymatic genes annotated as anthocyanin biosynthesis varied from 108 to 165 (49%-58%), which is considerably higher than the 56 genes (41%) annotated in *A. thaliana* and 93 genes (38%) in *A. chinensis* (Supplementary Table 3). Conversely, the number of chlorophyll and carotenoid biosynthesis genes showed a small variation among the seven *Rhododendron* species, ranging from 48 to 66 genes (Supplementary Table 3). The gene families of anthocyanin biosynthesis were generally larger than that of the carotenoid- and chlorophyll biosynthesis (Fig. 4a and Supplementary Table 3).

Further investigation revealed that the expansion of these pigment biosynthesis gene families was resulted from gene duplication, notably tandem duplication (TD) and proximal duplication (PD). The number of TD and PD genes related to anthocyanins in the seven *Rhododendron* species ranged from 49 to 95, accounting for 38%-58% of all anthocyanin-related genes, which is significantly higher than that observed in *A. thaliana* (30%) and *A. chinensi* (27%) (Fig. 4b, Supplementary Tables 4 and Supplementary Table 5). While TD and PD genes represented 16% − 29% in the carotenoid-related gene sets, and even lower proportions in the chlorophyll-related gene sets, with only 10% − 26% (Fig. 4b, Supplementary Table 5). These findings suggest that TD and PD genes have driven the expansion of the gene families of pigment biosynthesis which may associate with the distinct flower color diversity among *Rhododendron*.

### TEs’ potential role in gene duplication and pseudogenization related to flower color transition among *Rhododendron* species

We further investigated the pseudogenization of genes from three pigment biosynthetic pathways in seven *Rhododendron* species (Fig. 4c and Supplementary Table 6). Few pseudogenes were found in the carotenoid and chlorophyll biosynthesis, with only 3 to 10 pseudogenes identified across the seven *Rhododendron* species **(**Fig. 4c**)**. In contrast, a large number of pseudogenes were discovered in the anthocyanin biosynthetic gene families, particularly in *R. henanense* and *R. prattii***(**Fig. 4c**)**. This pseudogenization phenomenon led to a significant contraction of the anthocyanin biosynthetic gene family of *R. henanense* and *R. prattii*, which is noteworthy as flowers of both two species are in white without anthocyanin pigmentation. *R. molle* exhibited the second highest number of pseudogenes, with 26 pseudogenes in the anthocyanin gene families **(**Fig. 4c**)**. On the other hand, *R. simsii*, *R. ovatum*, *R. ripense*, and *R. griersonianum* had a relatively small number of pseudogenes in the anthocyanin gene families, and their flowers display a range of red color **(**Fig. 4c**)**. This suggests that the number of encoded genes in the anthocyanin gene families has contracted in the yellow-flowered *Rhododendron* species, *R. molle*, relative to red-flowered species.

Investigation of the association of LTR-RTs and gene duplication and loss provides valuable insights. Within the Chalcone synthase (CHS) family, a relatively high proportion of duplications and pseudogenizations were observed, which may contribute mostly to the expansion of CHS family (Supplementary Tables 8 and Supplementary Fig. 7). CHS is the first enzyme in flavonoid biosynthesis and a key enzyme in anthocyanin synthesis. We identified a conserved TD and PD CHS gene cluster present in all *Rhododendron* species (Supplementary Fig. 8 and Supplementary Table 7). The variation in gene number within the cluster may be associated with specific gene duplications and loss (pseudogenization) events. One possible mechanism for the formation of TD and PD and pseudogenes is through the insertion of TEs, as we found a substantial number of LTR-RT insertions upstream, downstream, and within the CHS genes, indicating their close association with the formation of TD/PD duplicates.

We selected the highest-count homologous chromosomal segments from the seven *Rhododendron* species for case studies. By constructing phylogenetic trees for these TD and PD genes and analyzing the homology of LTR-RTs surrounding the genes, we inferred the complex evolutionary relationships among genes in *Rhododendron* species (Supplementary Fig. 9 and Supplementary Table 7): (1) A gene that is completely conserved among species. For example, a gene is present in all seven *Rhododendron* species containing a conserved LTR-RTs element (00000757 L unknown) (Fig. 5a), implying that this gene originated in the ancestor of *Rhododendron*; (2) A gene that is conserved in specific species. For example, three *Rhododendron* species from the *Tsutsusi* subgenus show a specific synteny relationship (Fig. 5b), while missing in others, indicating the origin of this gene in the ancestor of *Tsutsusi*. In *R. prattii* and *R. henanense*, a gene with conservation was found, and both contained a conserved LTR-RTs element (TE_00010703 LTR/*Copia*) (Fig. 5b), suggesting that this gene originated before the divergence of *R. prattii* and *R. henanense*; (3) Clusters of multiple duplicated genes are conserved among rhododendrons. For example, two CHS TD/PD genes show conservation in all seven *Rhododendron* species (Fig. 5c), and three genes show specific synteny in the *Tsutsusi* subgenus (Fig. 5d), indicating gene duplications occurring prior to the differentiation of the entire genus *Rhododendron* and the *Tsutsusi* subgenus. (4) Pseudogenization is associated with the loss of family members. A gene showing synteny in the *Tsutsusi* and *Pentanthera* subgenera has undergone loss-of-function variation and become a pseudogene in the *Hymenanthes* subgenus (Fig. 5f). Moreover, some genes have undergone amplification in specific species and subsequently became pseudogenes (Fig. 5e and g).

The complexity of gene duplication and pseudogenization may be attributed to the movement of LTR-RTs. Compared to other *Rhododendron* species, *R. prattii* exhibited a high frequency of gene duplication events (Fig. 4b). Additionally, *R. prattii* and *R. henanense* contained eight and four pseudogenes, respectively, within this gene cluster (Supplementary Tables 7 and Fig. 4c). Except for *R. simsii*, which did not exhibit pseudogenization in homologous chromosomes, only 1–2 CHS genes underwent pseudogenization in the other *Rhododendron* species (Supplementary Tables 7 and Fig. 4c). Correspondingly, within this gene cluster region, *R. prattii* had the highest content and number of LTR-RTs (Supplementary Fig. 10).

## Discussion

Transposable elements (TEs) influence genome size and chromosome arrangements significantly, particularly in species where TEs comprise a substantial portion of the DNA [45]. In this study, we performed a whole-genome analysis to identify and compare repetitive sequences in the genomes of seven *Rhododendron* species. Our findings revealed that TEs accounted for approximately half of the genome size in published *Rhododendron* genomes. Changes in the number of repetitive elements are frequently cited as the cause of genome size variance amongst species with the same chromosome number [46, 47]. This phenomenon was confirmed in these seven *Rhododendron* species studied here, where the proportion of TEs positively correlated with genome size and agreed with their phylogenetic relationships. Notably, long terminal repeat retrotransposons (LTR-RTs) had the highest abundant among all TEs, aligning with previous studies suggesting that LTR-RTs are key factors influencing plant genomes [48, 49]. The transposition mechanism of LTR-RTs allows them to influence genome size and structure during transposition [50–52]. By quantifying the rates of LTR-RTs creation and removal, we were able to explain how transposons impact genome size. It was revealed in the present study that the proportion of novel LTR-RTs unique to the seven *Rhododendron* species was very low, suggesting similar evolutionary histories among them.

We also estimated the insertion times of *Gypsy* and *Copia* elements and found recent bursts in both types of LTR-RTs. Previous studies have shown that the repetitive nature of many plant genomes is determined by a few highly abundant LTR-RT families. For instance, the *Gypsy* superfamilies dominates the genomes of tea [53] and *Arabidopsis thaliana* [52], while the Copia retrotransposon family prevails within the grapevine genome [54]. In *Rhododendron*, *Gypsy*-type LTR-RTs had a significant role in recent dynamic changes in genome size, especially in the *Hymenanthes* and *Pentanthera* subgenera. Also, the Retand element within the *Gypsy* family stands out as a key element driving these changes. This suggests that the distinct interspecific variations in the repetitive sequence of *Gypsy*-Retand contribute to the relative expansion of genome size in the *Hymenanthes* and *Pentanthera* subgenera. Interestingly, within the *Gypsy* family, the Retand element appears to be particularly responsible for this phenomenon. These findings underscore the crucial role of LTR-RTs as driving factors behind species divergence and diversification.

The regions surrounding TEs vary extensively, and represent the unstable and species-specific regions of genomes [55]. Our comparative genomic analysis identified inter-specific chromosomal structural variations at the whole-genome level. Although there are some syntenic and conserved regions among these species, we discovered that variances still occur to varied degrees. The higher accumulation of LTR-RTs around inversion regions (INV) and species-specific regions (SPE), particularly of the *Gypsy*, demonstrates that the insertion of specific clade LTR-RTs may be one of the causes of interspecific variation in *Rhododendron* species. Furthermore, our study found that genes located in specific regions of the *Rhododendron* genomes have lower expression levels compared to genes located in syntenic regions. We also observed that genes associated with anthocyanin and carotenoid biosynthesis in the specific regions demonstrated significantly reduced expression compared to genes related to chlorophyll. This is in line with previous findings in *A. thaliana* and soybean, where genes containing TEs were found to have slightly lower average expression levels [56–58]. This suggests that TE insertions, which are more abundant in specific regions, may influence the expression of certain genes during the flowering process of rhododendron. However, more research is needed to investigate the underlying mechanisms.

In *Rhododendron* species, flower color is a highly important and extensively studied trait, largely determined by the type and quantity of pigments present in the petals [59]. By annotating the anthocyanin, chlorophyll, and carotenoid metabolic pathways, we discovered that *Rhododendron* species possess the highest number of genes associated with anthocyanin biosynthesis. Furthermore, rhododendrons exhibit a higher percentage of anthocyanin-related genes compared to *A. thaliana* and sunflower, corroborating previous research that highlights the primary role of anthocyanins in influencing flower color in *Rhododendron* species [2, 9]. In the present study, we identified a significant number of tandem duplication (TD) and proximal (PD) duplication in anthocyanin biosynthetic gene families across the seven rhododendrons. This finding suggests that TD and PD events distinctly contribute to the expansion of anthocyanin-related genes in *Rhododendron*. At the gene family level, we examined the CHS gene family to better understand the patterns of gene gain and loss differentiation between species. Numerous collinearity relationships were observed among these TD and PD genes within the CHS gene family among *Rhododendron* species, along with a substantial presence of homologous LTR-RTs near these genes. TEs, particularly LTR-RTs, are a potential mechanism for generating TD and PD [60], and our results strongly indicate a close association between TD and PD in anthocyanin biosynthetic genes and LTR-RT elements.

Repetitive sequences, as a powerful driving force behind genomic variation, can actively or passively induce ontogenetic variations during gene birth and elimination processes [61]. This includes gene duplications and losses. Pseudogenization, which are gene remnants that have lost their function, represent an important pattern of gene loss [62, 63]. In our study, we focused on analyzing pseudogenization of genes involved in anthocyanin, chlorophyll, and carotenoid biosynthesis. The results revealed a higher prevalence of pseudogenization in the anthocyanin-related gene families of *R. henanense*, *R. prattii*, and *R. molle*. Interestingly, these three *Rhododendron* species exhibit white and yellow flower colors, whereas species with red flowers, such as *R. simsii*, *R. ovatum*, *R. ripense*, and *R. griersonianum*, showed lower degrees of pseudogenization. This finding further supports the hypothesis that anthocyanins may play a crucial role in shaping flower color in *Rhododendron* species. Overall, our study identifies both gene duplications and losses as common occurrences in the anthocyanin biosynthesis gene families. We speculate that this phenomenon may contribute to the significant diversity and variation in flower color observed in *Rhododendron*, with anthocyanins serving as important effectors in this process.

## Conclusion

In this study, we collated genomic data from seven high-quality, published *Rhododendron* species. This data was used to identify repetitive sequences and conduct an inter-species comparative genomic analysis. Transposable elements (TEs) were shown to comprise half of the genome size within the *Rhododendron* genus. Predominantly, long terminal repeat retrotransposons (LTRs) demonstrated a consistent correlation with genome size and phylogenetic relationships. This commonality can be attributed to the production and elimination rates of these elements. Species-specific regions (SPE), inversion regions (INV), and species-syntenic regions (SYN) were identified in the genomes of the seven *Rhododendron* species. Different proportions of LTRs were also identified. The prevalent occurrence of *Gypsy*, a principal LTR-RTs element, suggests it may play a key role in driving inter-species structural variations. In addition, we probed the mechanisms by which LTRs affect pigment-related genes at a whole-genome level, using members of the CHS gene family as examples. Our study found that gene duplication, loss and corresponding expression associated with flower color could be influenced by TEs. Notably, the anthocyanin-related gene family, which impacts flower color, is characterized by numerical amplifications and pseudogenization in the *Rhododendron* genus. Our research underscores the pivotal role of TEs in dictating the complexity and floral color diversity of the *Rhododendron* genome, providing valuable insights into how TEs drive inter-species variations.

## Supplementary Information

Supplementary Material 1.

Supplementary Material 2.

## Data Availability

The datasets generated and/or analysed during the current study are available in the Zenodo repository (https://zenodo.org/records/15358092?preview=1&token=eyJhbGciOiJIUzUxMiJ9.eyJpZCI6ImJkNWRmNWQ0LTFkY2ItNDE5Mi1hNjIzLTU3ODQ5MTNjYjRmMSIsImRhdGEiOnt9LCJyYW5kb20iOiJmYjU3NGE1YTE2YTc4YjI4OTUyNmQwYzcwMzVmNWUwZSJ9.yckw_2q6oFJF9MLTBcCRvOKHohdkMU7kSvAmb90MD1AwsmAIt8atAhp-UfMdF-j5dAvH1XF04SkFxD9o7lRM2Q). The publicly available genomic data utilized in this study include: *R. molle* (Bioproject accession: PRJNA804375), *R. simsi* (Bioproject accession: PRJNA588298), *R. griersonianum* (Bioproject accession: PRJNA659608), *R. ovatum* (Bioproject accession: PRJNA671625), *R. henanense* (Bioproject accession: PRJNA656593), *R. williamsianum* (Bioproject accession: PRJNA432092), and *R. delavayi* (Bioproject accession: PRJNA361437), all of which were downloaded from NCBI. The genomic data for *R. prattii* (GWH: GWHBHLU00000000) were obtained from in the National Genomics Data Center, and .the genomic data for *R. ripense* (DDBJ: DRA012151) were obtained from in the Sequence Read Archive (DRA) of the DNA Data Bank of Japan (DDBJ).

## Abbreviations

TEs: transposable elements
LTR-RTs: long terminal repeat retrotransposons
SYN: species-syntenic regions
INV: inversion regions
SPE: species-specific regions
TD: tandem duplication
PD: proximal duplication
