# Supplementary material for: A pangenome insight into the genome divergence and flower color diversity among Rhododendron species
Source: BMC Genomics. 2026 Jan 6;27:101. doi: 10.1186/s12864-025-12461-5 (PMC12837525; doi:10.1186/s12864-025-12461-5)
Supplement: Supplementary file 2 — Supplementary Material 2. [file 12864_2025_12461_MOESM2_ESM.docx]

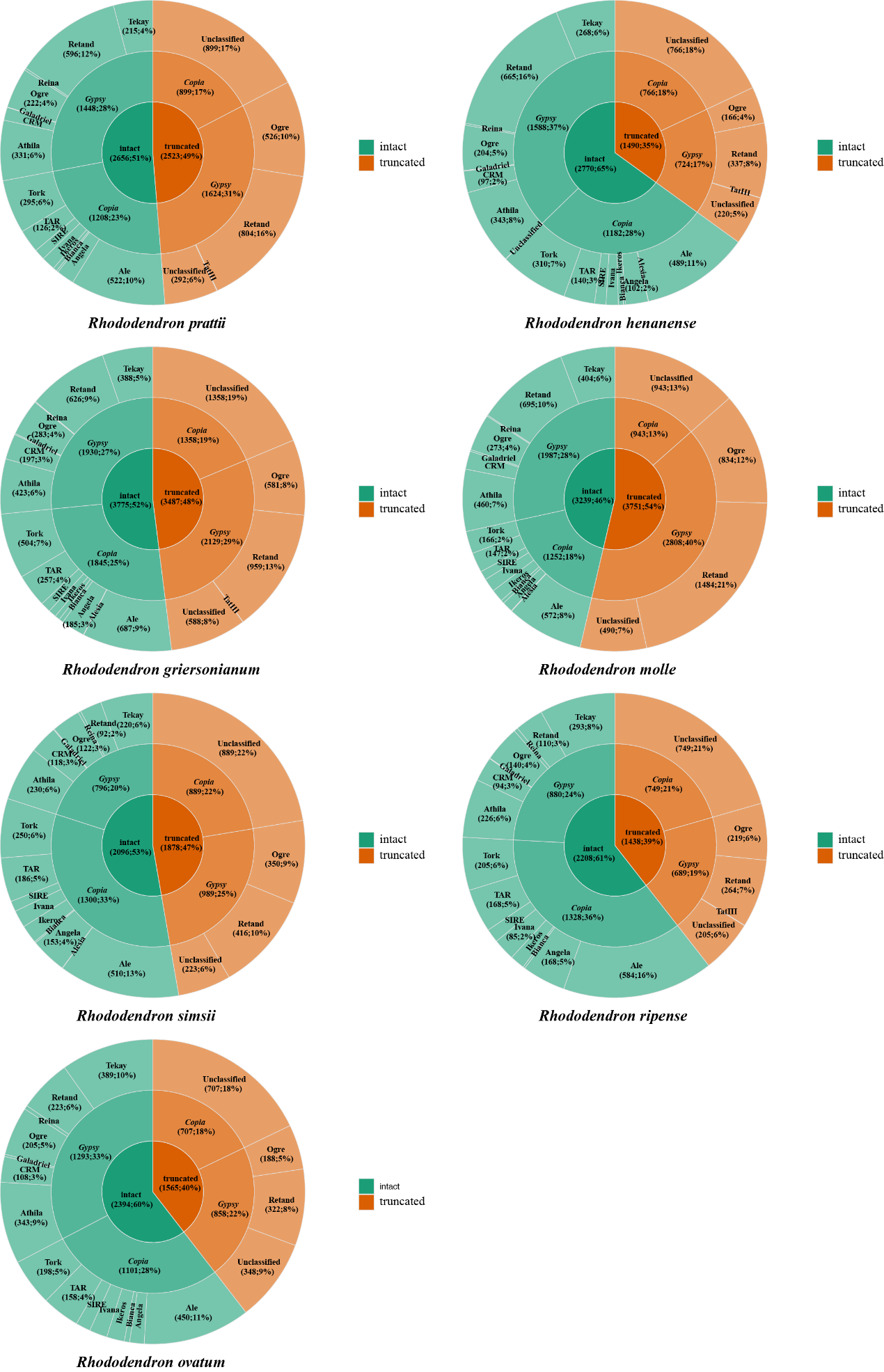


**Supplementary Figure 1** Count proportions of the LTR-RTs.


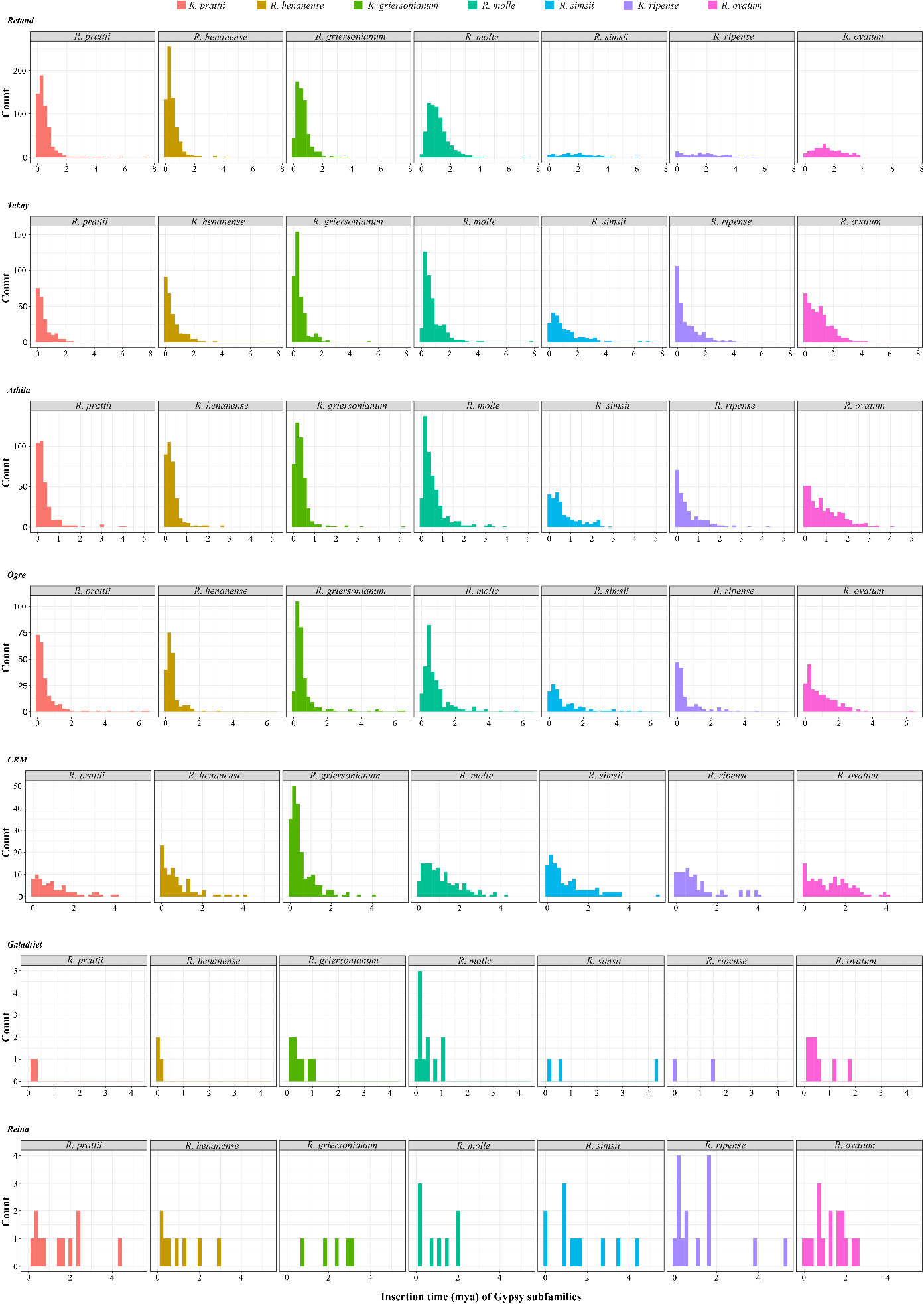


**Supplementary Figure 2** Insertion time of the Gypsy element


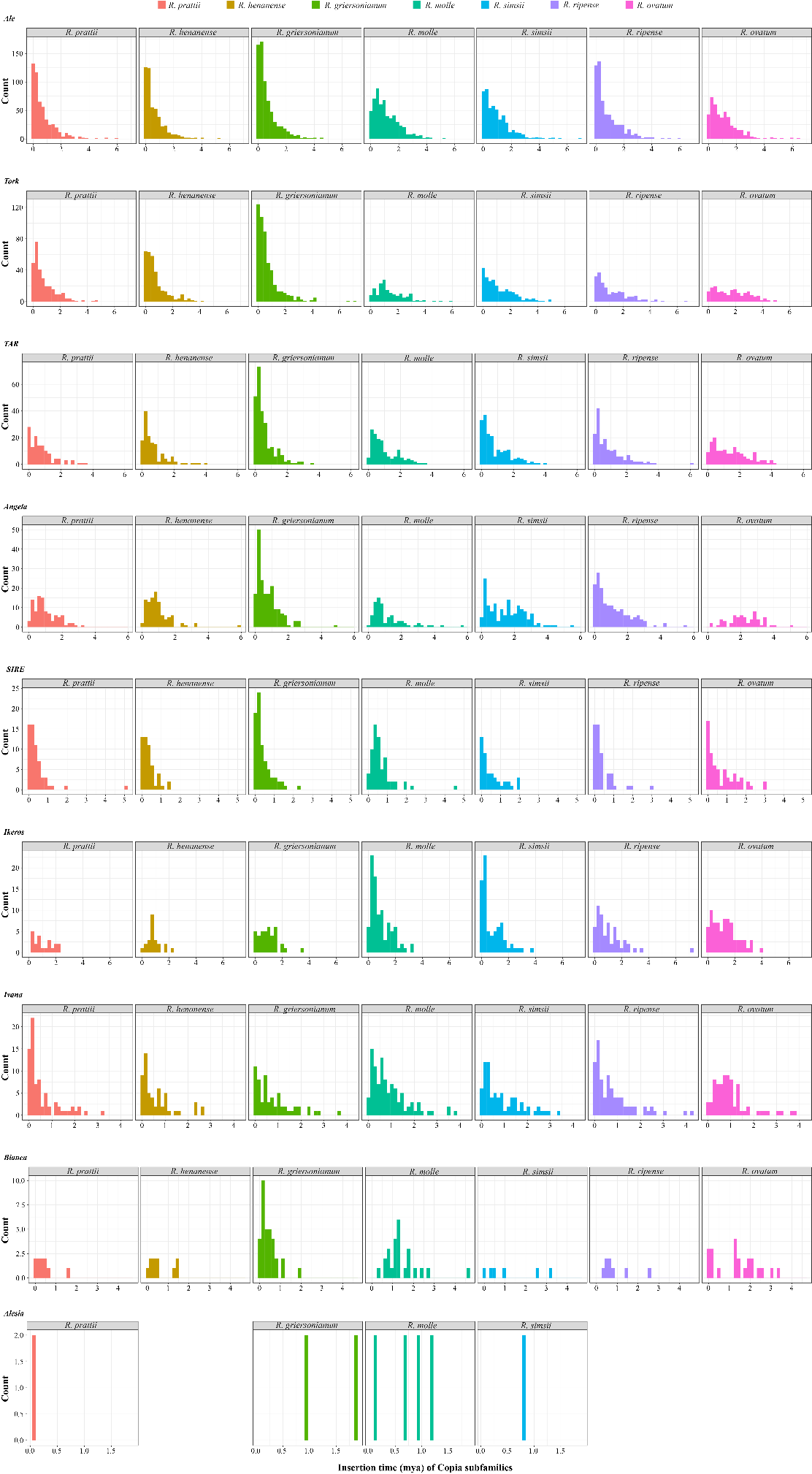


**Supplementary Figure 3** Insertion time of the Copia elements.


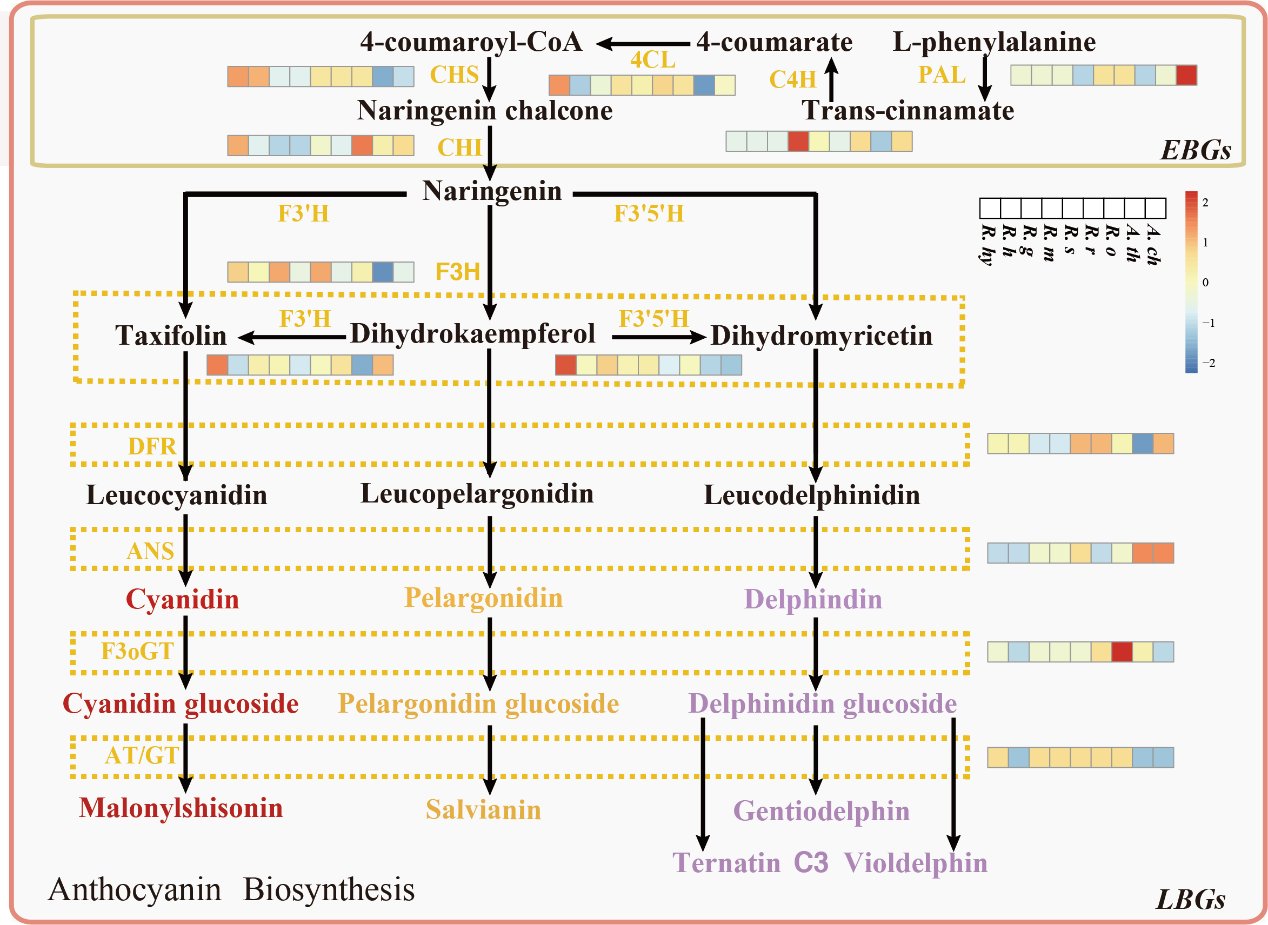
 **Supplementary Figure 4** Statistical analysis of the biosynthesis pathways of anthocyanins.


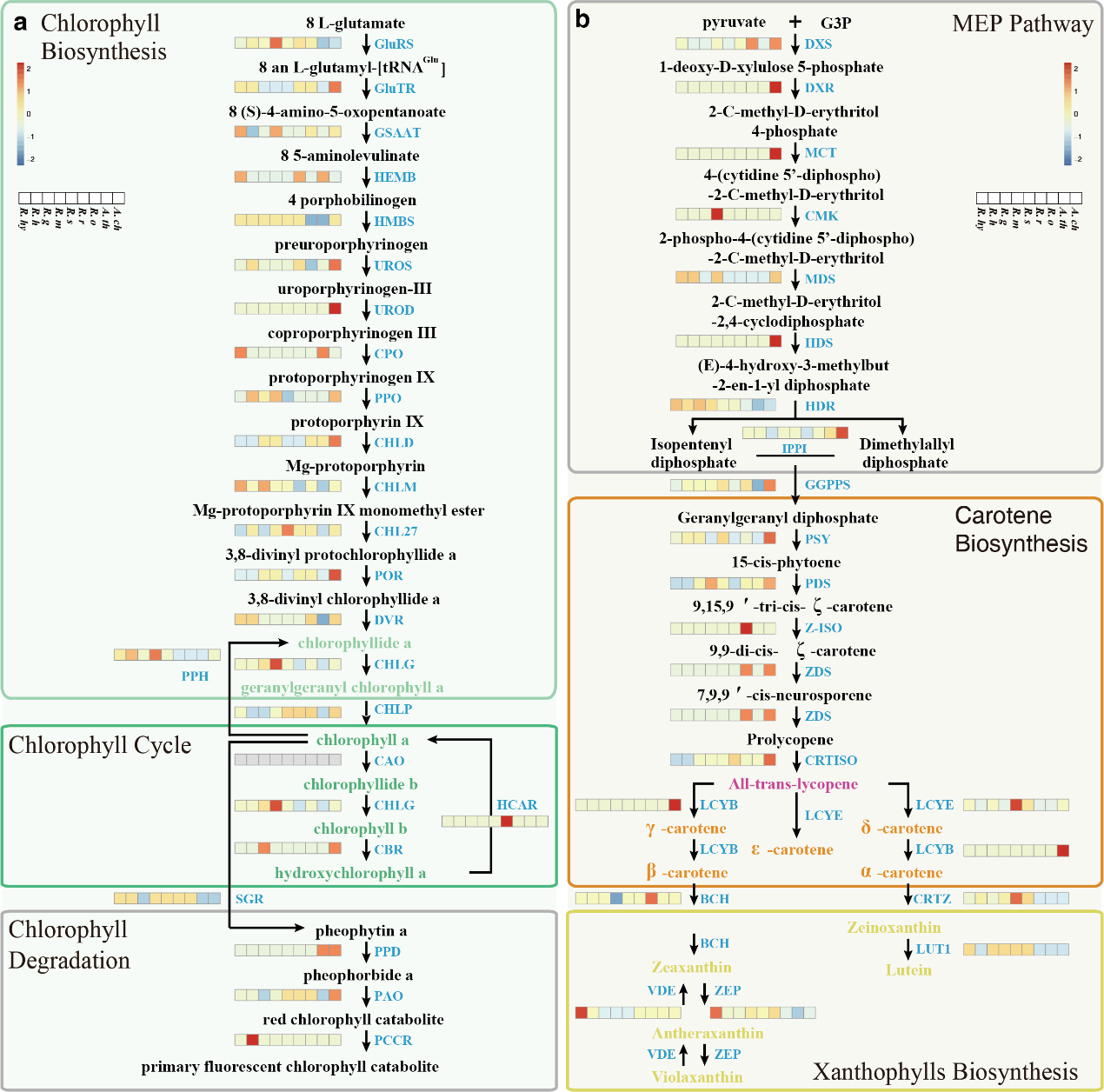


**Supplementary Figure 5** Statistical analysis of the biosynthesis pathways of chlorophylls, and carotenoids.


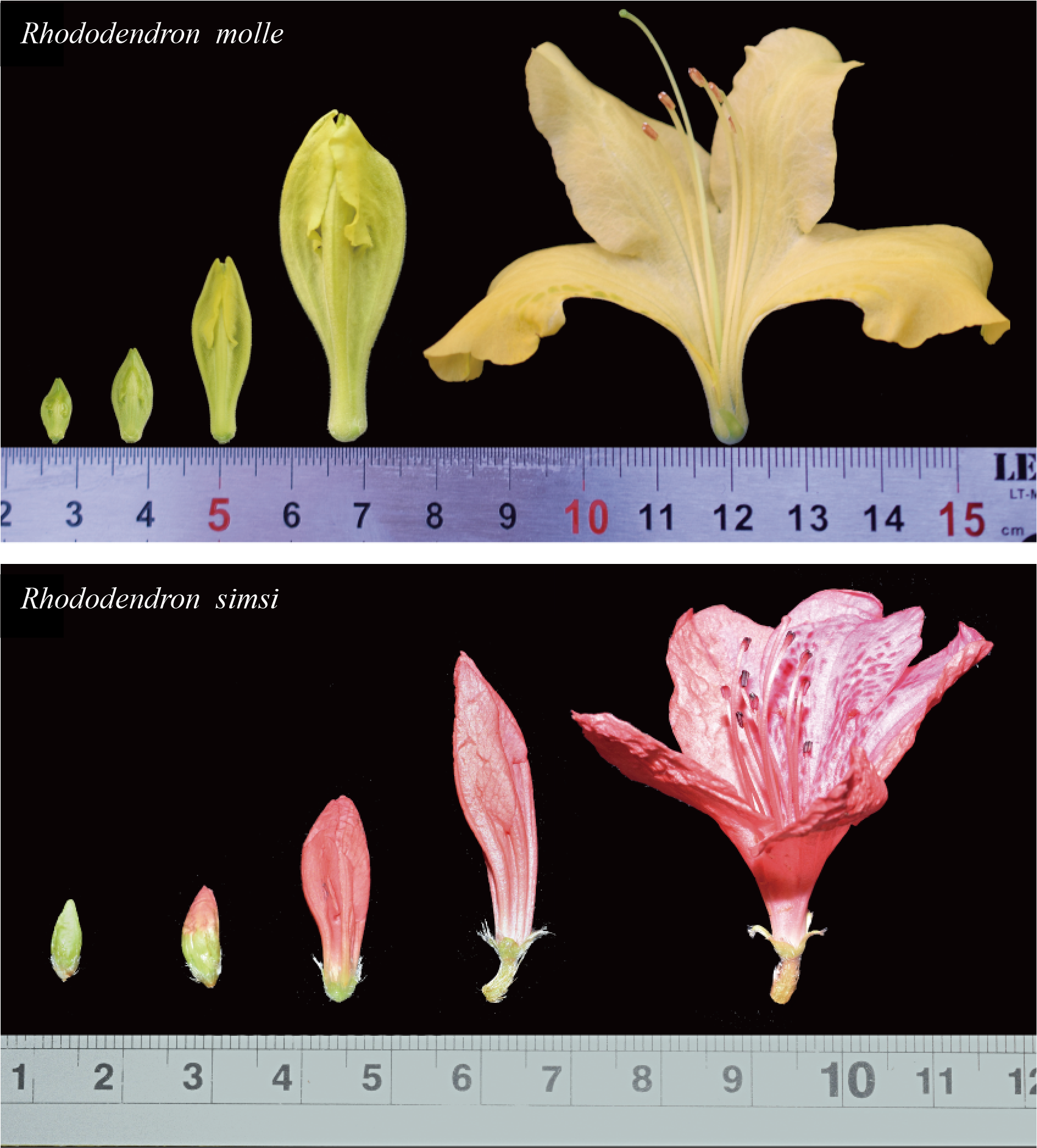


**Supplementary Figure 6** Five flower developmental time points (T1-T5) (*R. molle* [10] and *R. simsii* [10]).


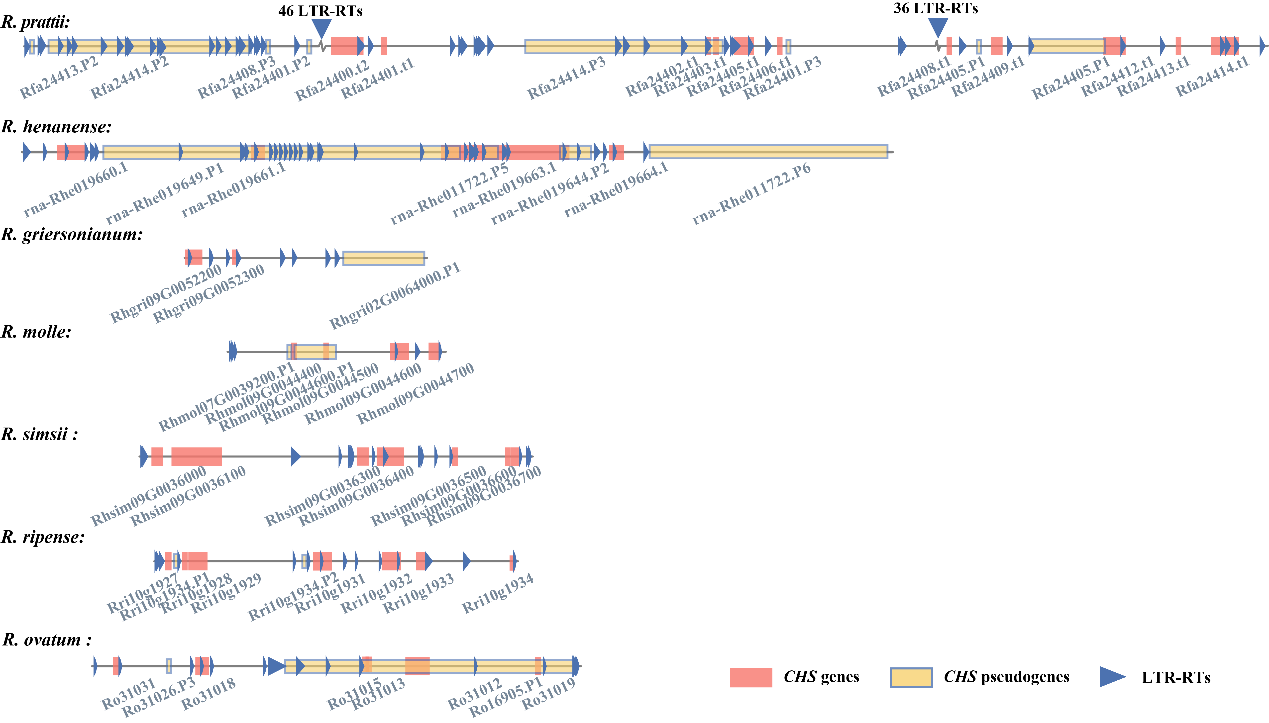


**Supplementary Figure 7** The proportion of TD and PD genes associated with the anthocyanin biosynthetic pathway genes.


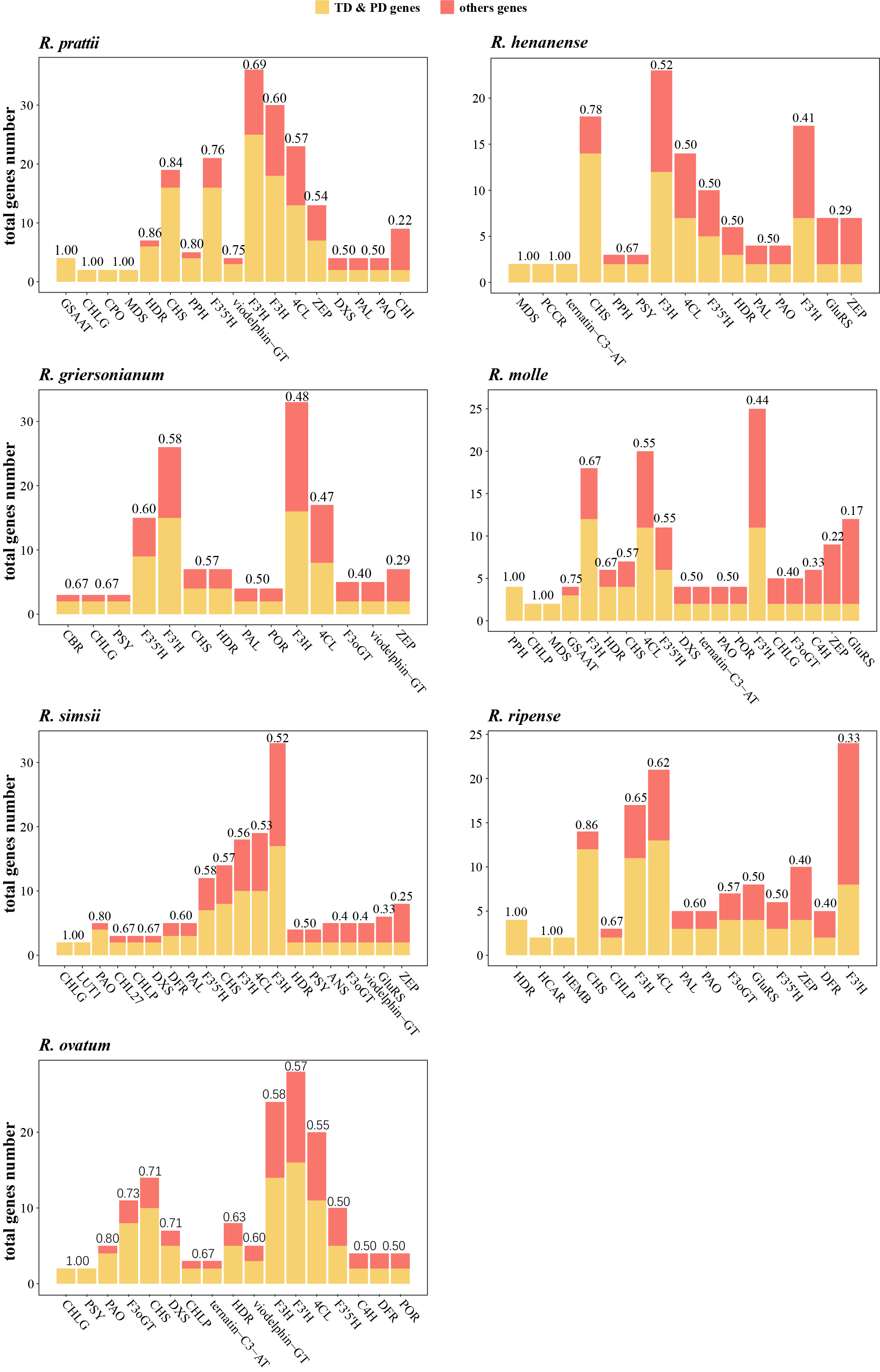


**Supplementary Figure 8** The TD and PD genes and pseudogenes of the CHS gene family within a specific chromosomal region in 7 rhododendrons.


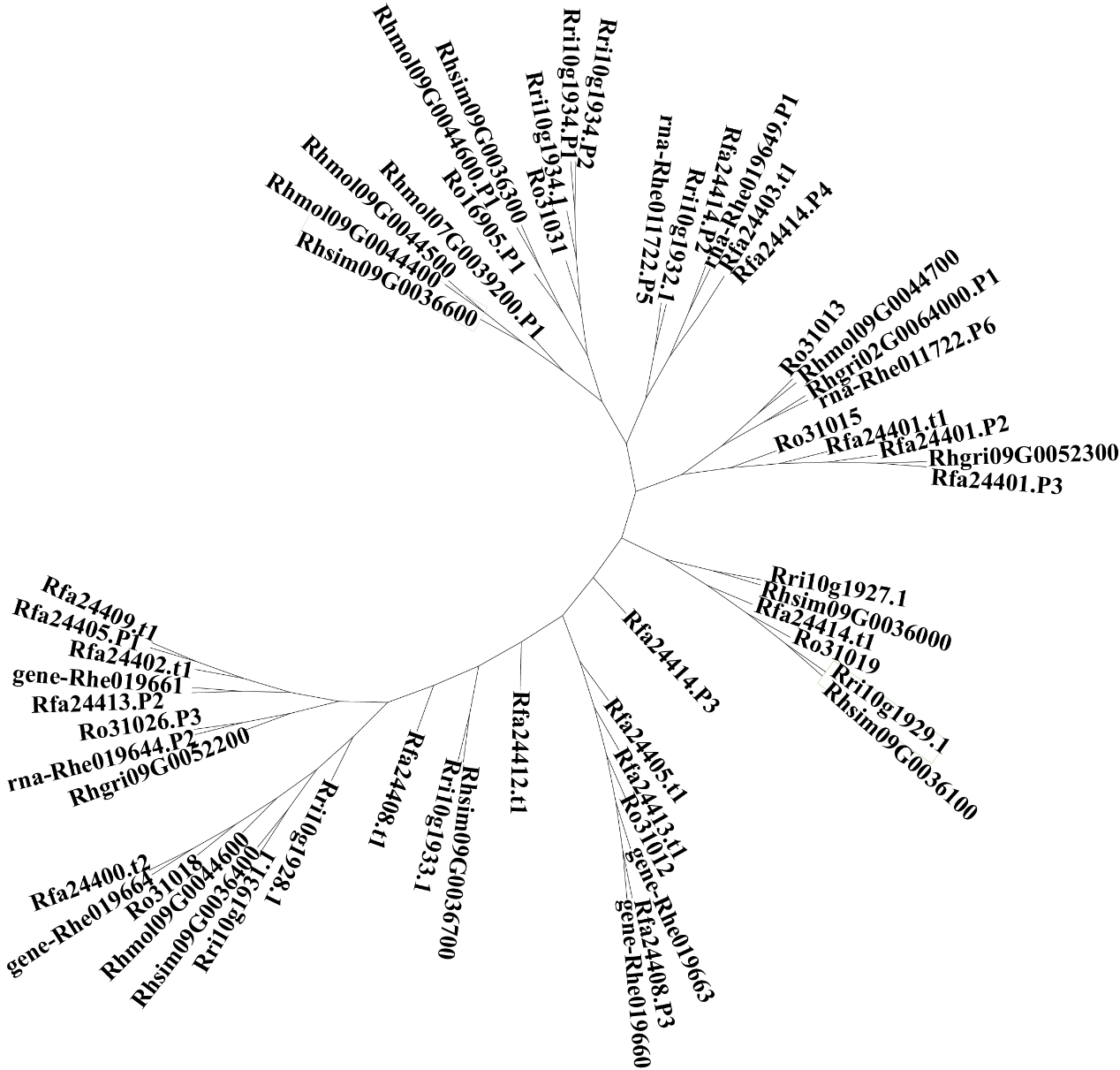


**Supplementary Figure 9** Phylogenetic Tree of TD and PD Genes and pseudogenes in the CHS Gene Family.


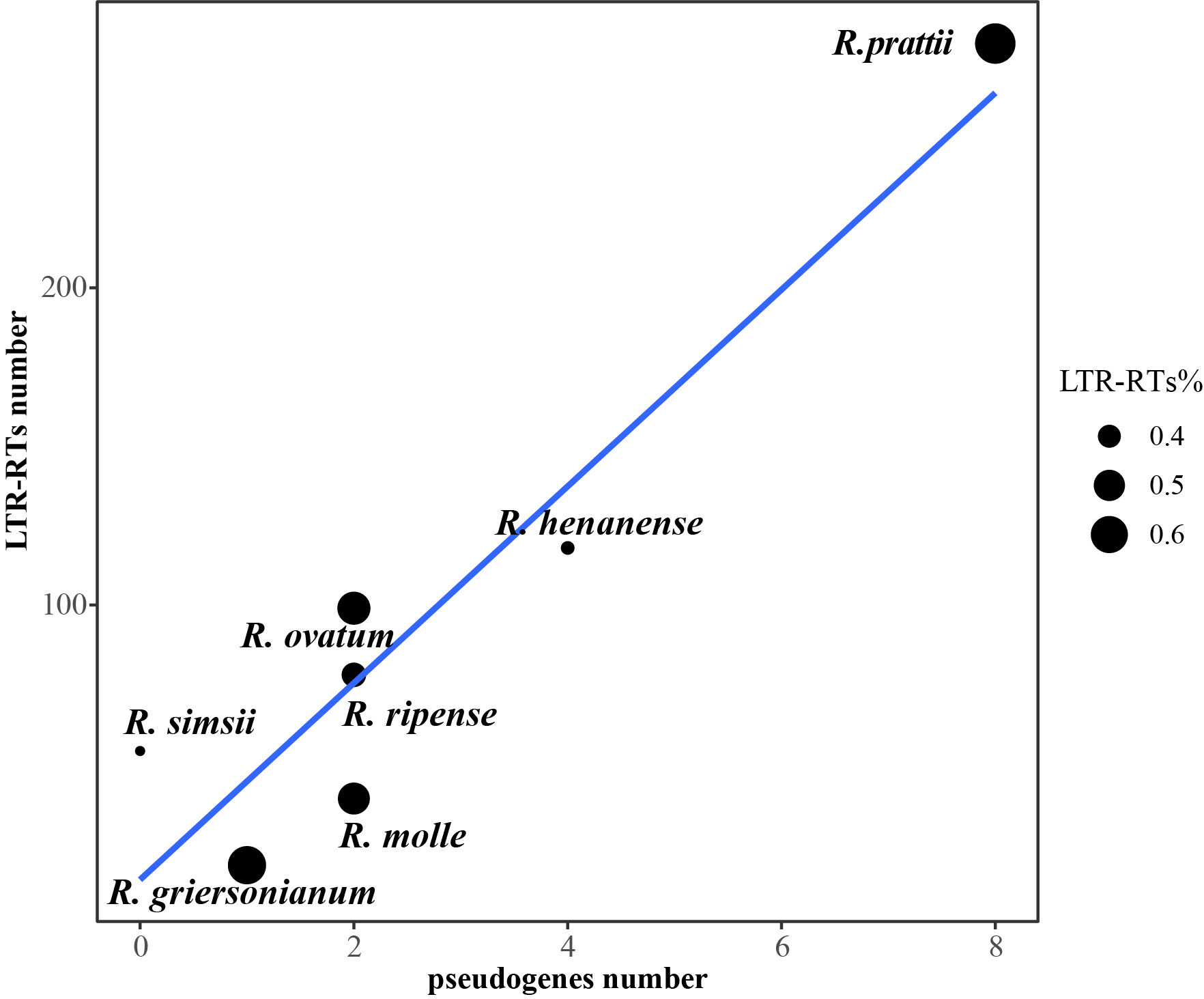


**Supplementary Figure 10** The correlation between pseudogene abundance and LTR-RTs.
